# The Antioxidant Cofactor Alpha-Lipoic Acid May Control Endogenous Formaldehyde Metabolism in Mammals

**DOI:** 10.3389/fnins.2017.00651

**Published:** 2017-12-01

**Authors:** Anastasia V. Shindyapina, Tatiana V. Komarova, Ekaterina V. Sheshukova, Natalia M. Ershova, Vadim N. Tashlitsky, Alexander V. Kurkin, Ildar R. Yusupov, Garik V. Mkrtchyan, Murat Y. Shagidulin, Yuri L. Dorokhov

**Affiliations:** ^1^Department of Genetics and Biotechnology, N. I. Vavilov Institute of General Genetics, Russian Academy of Science, Moscow, Russia; ^2^Department of Chemistry and Biochemistry of Nucleoproteins, A. N. Belozersky Institute of Physico-Chemical Biology, Lomonosov Moscow State University, Moscow, Russia; ^3^Faculty of Chemistry, Lomonosov Moscow State University, Moscow, Russia; ^4^Academician V. I. Schumakov Federal Research Center of Transplantology and Artificial Organs, Moscow, Russia

**Keywords:** endogenous methanol, formaldehyde, alpha-lipoic acid, glutathione, alcohol dehydrogenase, aldehyde dehydrogenase 2, brain, hippocampus

## Abstract

The healthy human body contains small amounts of metabolic formaldehyde (FA) that mainly results from methanol oxidation by pectin methylesterase, which is active in a vegetable diet and in the gastrointestinal microbiome. With age, the ability to maintain a low level of FA decreases, which increases the risk of Alzheimer's disease and dementia. It has been shown that 1,2-dithiolane-3-pentanoic acid or alpha lipoic acid (ALA), a naturally occurring dithiol and antioxidant cofactor of mitochondrial α-ketoacid dehydrogenases, increases glutathione (GSH) content and FA metabolism by mitochondrial aldehyde dehydrogenase 2 (ALDH2) thus manifests a therapeutic potential beyond its antioxidant property. We suggested that ALA can contribute to a decrease in the FA content of mammals by acting on *ALDH2* expression. To test this assumption, we administered ALA in mice in order to examine the effect on FA metabolism and collected blood samples for the measurement of FA. Our data revealed that ALA efficiently eliminated FA in mice. Without affecting the specific activity of FA-metabolizing enzymes (ADH1, ALDH2, and ADH5), ALA increased the GSH content in the brain and up-regulated the expression of the FA-metabolizing *ALDH2* gene in the brain, particularly in the hippocampus, but did not impact its expression in the liver *in vivo* or in rat liver isolated from the rest of the body. After ALA administration in mice and in accordance with the increased content of brain *ALDH2* mRNA, we detected increased ALDH2 activity in brain homogenates. We hypothesized that the beneficial effects of ALA on patients with Alzheimer's disease may be associated with accelerated ALDH2-mediated FA detoxification and clearance.

## Introduction

In mammals, the oxidation of metabolic short-chain alcohols, such as methanol (MeOH) and ethanol (EtOH), occurs with the participation of enzymes from the class of alcohol dehydrogenase 1 (ADH1) (Cederbaum, 2012; Wang et al., 2012; Edenberg and Foroud, 2013; Dorokhov et al., 2015). MeOH produced by pectin methylesterases (PME) of the gastrointestinal microbiome or by plant PMEs consumed with vegetable food (Dorokhov et al., 2012, 2015; Komarova et al., 2014; Shindyapina et al., 2014) is invariably converted by the ADH1 enzyme to formaldehyde (FA) in mammalian organisms. MeOH is not the only source of metabolic FA; it may also be formed as a result of (a) semicarbazide-sensitive amine oxidase-mediated oxidative deamination of the methylamine derived from creatinine (Charlton and Mack, 1994; Lee et al., 2008) and (b) chromatin structure remodeling in the reaction of methyl groups removal from lysine residues in histones, which is catalyzed by lysine-specific demethylase 1 and JmjC domain-containing histone demethylases (Tsukada et al., 2006; Cloos et al., 2008; Hou and Yu, 2010; Walport et al., 2012; Liu et al., 2013).

FA appears to be the relevant agent in neurodegeneration (below); its oxidation to formic acid constitutes detoxification in this context. FA is converted to formic acid by the participation of enzymes mainly from the class of aldehyde dehydrogenase 2 (AlDH2) (Sládek, 2003; Sophos and Vasiliou, 2003). The oxidation of FA also occurs by the participation of ADH5 or the glutathione-dependent χ-ADH FA dehydrogenase (FDH) enzyme (Tulpule et al., 2013). At the same time, it is known that catalase (CAT) (Cederbaum and Qureshi, 1982; Deng and Deitrich, 2008) and cytochrome P450 (CYP2E1) (Coon and Koop, 1987; Caro and Cederbaum, 2004; Wallage and Watterson, 2008) are included in the process of alcohol metabolism at high alcohol concentrations. It is important to note that in the tissues of the brain and liver, the regulation of the levels of MeOH occurs in different ways. The ADH1 enzyme oxidizes most of the MeOH in the human liver, while in brain cells, its activity is close to zero (Julià et al., 1987; Galter et al., 2003; Tulpule and Dringen, 2013). Apparently, the low activity of the ADH1 enzyme in human brain cells acts as a mechanism of protection against the harmful effects of FA on neurons (Tulpule and Dringen, 2013).

Chronic exposure to FA is known to cause acute health problems (Tang et al., 2009) related to amyloid aggregation (Chen et al., 2007), tau protein aggregation and hyperphosphorylation *in vitro* and *in vivo* (ENCODE Project Consortium et al., 2007; Liu et al., 2013; Su et al., 2016). It has been suggested that FA toxicity is related to the main hallmarks of age-related neuronal damage and Alzheimer's disease pathology (Tong et al., 2011, 2013a,b, 2017; Liu et al., 2013). The elevated levels of FA found in Alzheimer's disease (Tang et al., 2013a,b; Tong et al., 2013a, 2017) may play important roles in β-amyloid (Aβ) aggregation and cerebral amyloid angiopathy (Li et al., 2016b).

An understanding of the mechanisms of FA metabolism and the ability to maintain a low level of its content in the human body open up the possibility of its control, reducing the risk of age-related neuronal damage and Alzheimer's disease pathology. In principle, there are two key strategies to balance the endogenous FA concentration: (i) inhibition of ADH1-mediated FA synthesis and (ii) stimulation of FA clearance through ALDH2 and ADH5 activation. The reality of the latter method was confirmed by the high detoxifying ability of *ALDH2* transgenic mice (Doser et al., 2009). Current knowledge of the factors that contribute to maintaining a low level of mammalian FA content is limited; however, one should take a closer look at the ways to alleviate the lives of patients suffering from Alzheimer's disease by potentially reducing the content of FA (Venigalla et al., 2016). The list of nutraceuticals with antioxidant, neuroprotective, and cognition-enhancing properties contains alpha lipoic acid (ALA, 1,2-dithiolane-3-pentanoic acid) (Moura et al., 2015; Cronan, 2016), which has the potential for Alzheimer's disease prevention and treatment (Venigalla et al., 2016). There is evidence that ALA delays the aging process in the brain and improves brain function and memory. Studies on rat aging have suggested that ALA usage results in improved memory performance and delayed structural mitochondrial decay (Ames and Liu, 2004). Historically, the ability of ALA to ameliorate vitamin C and E deficiencies in laboratory guinea pigs and rats, respectively, first suggested that ALA functions as an antioxidant (Rosenberg and Culik, 1959). Oxidized and reduced (dihydrolipoic acid, DHLA) forms of ALA have been found to chelate heavy metals (Ou et al., 1995), scavenge various reactive oxygen species (ROS) (Packer et al., 1995), and affect antioxidant gene expression (Shay et al., 2009). Although only the R-ALA stereoisomer is found as a cofactor in ALA-containing enzymes, both enantiomers are effective scavengers of various ROS when they are administered exogenously (Bingham et al., 2014). DHLA can regenerate oxidized antioxidants, such as ascorbate (vitamin C), glutathione, coenzyme Q10, and vitamin E, thus reactivating them (Constantinescu et al., 1995). ALA also increases the levels of the most important water-soluble endogenous antioxidant, glutathione (GSH), *in vivo* and *in vitro* (Busse et al., 1992; Podda et al., 1994; Han et al., 1997; Sen et al., 1997). Glutathione reacts with FA, forming S-formylglutathione, which in turn is readily oxidized by ADH5. However, the possible participation of ALA in the metabolism of endogenous FA is not only based on its ability to increase GSH. Recent studies have shown that incubation with DHLA increases the activity of another FA-metabolizing enzyme—ALDH2 (He et al., 2012; Li et al., 2013; McCarty, 2013; Dudek et al., 2014)—by the reduction of disulphide bonds between the cysteines in the active site (Muñoz-Clares et al., 2017). A course of ALA injections resulted in elevated ALDH2 activity in human serum with coronary disease and in the gastric mucosa of rats treated with ethanol (Li et al., 2013, 2016a).

Thus, it can be assumed that ALA can perform the function of a regulator of ALDH2 activities in mammals and can thus control FA metabolism. To test this assumption, we investigated the effect of ALA on the metabolism of FA in mice. Our results showed that ALA is able to decrease exogenous and metabolic MeOH and FA levels in mice. The mechanism of FA elimination involves up-regulation of the genes responsible for its metabolism (*ADH1, ALDH2, CAT, CYP2E1*) in the hippocampus and spleen and elevated enzymatic activity of ALDH2 in mouse brain.

## Materials and methods

### Materials

β-nicotinamide adenine dinucleotide, acetonitrile (HPLC grade) and Triton X-100 were purchased from PanReac AppliChem, Halt Protease Inhibitor Cocktail (100X) and 16% methanol-free formaldehyde from Thermo Fisher Scientific, TriReagent from MRC. Alda-1 was synthesized by Dr. A.V. Kurkin (Moscow State University, Russia). All other chemicals were from Sigma Aldrich (Vienna, Austria) if not specified otherwise.

### Animals and *in Vivo* treatment

Animal procedures were in accordance with Guide for the Care and Use of Laboratory Animals as adopted and promulgated by the U.S. National Institutes of Health and permission was granted by the ethical committee of the A. N. Belozersky Institute of Physico-Chemical Biology at Lomonosov Moscow State University, Moscow, Russia (protocol number 4 from May 12, 2016). All mice used in the study were female non-pregnant Balb/c mice with weights of 28–34 g. Liver perfusion was performed on Wistar male rats. The animals were fed a cereal-based diet consisting of 12.7% protein, 5.6% fat, and 54.1% carbohydrate with a total fiber content of 3.7%. The diet was supplemented with a vitamin-mineral premix according to the recommendation of the American Institute of Nutrition (AIN-93M diet). Mice were randomly assigned to 3 groups of five and were injected i.p. with 300 μl of normal saline or 4-MP (10 mg/kg) with corresponding volume of ALA vehicle in normal saline or a mix of ALA (20 mg/kg) and 4-MP (10 mg/kg) in normal saline. The same protocol was used for Alda-1 (8.5 mg/kg) treatment. For the treatment with ascorbic acid, mice were assigned to two groups of three and were injected i.p. with 300 μl of 4-MP (10 mg/kg) or the same volume of a mix of ascorbic acid (200 mg/kg) and 4-MP (10 mg/kg) in normal saline. Approximately 100 μl of 4-MP (10 mg/kg) in olive oil or a mix of 4-MP (10 mg/kg) and tocopherol (100 mg/kg) in olive oil were orally administered. Blood samples (50–100 μl), whole brain and liver were collected 90 min later. Liver and brain tissues were immediately flash-frozen in liquid nitrogen and homogenized, and 50–100 mg was transferred to 1–1.5 ml of TriReagent. Total RNA was extracted by TriReagent according to the manufacturer's protocol. Blood samples were incubated at 25°C for clot formation. Plasma was separated by centrifugation at 700 g for 20 min (4°C), transferred to sterile tubes and mixed with an equal volume of 10% trichloroacetic acid (TCA) for FA measurement or with an equal volume of acetonitrile supplemented with 1% Triton X-100 for MeOH measurement. The mixtures were incubated for 20 min on ice and were then centrifuged for 10 min at 16,000 g. Finally, the supernatant was transferred to sterile tubes and analyzed for MeOH and FA content by HPLC and gas chromatography (GC), respectively. Expression analysis was blinded for chemical injection and RNA extraction.

### Rat liver perfusion technique

All manipulations were carried out under sterile conditions, taking into account the aseptic and antiseptic requirements. Anaesthesia of experimental animals was carried out by injecting the solution Zoletil 50 (Virbac Sante Animale, France) into the abdominal cavity at a dosage of 7.5 mg/kg body weight. For the purpose of systemic heparinization, heparin was administered to the penile vein at a dose of 200 units. Then, median laparotomy was performed. Using a special cannula (Venflon 1.3 × 45 mm), the portal vein (*Vena portae*) was cannulated and an additional 150 units of heparin was added to the portal vein. Immediately after this, the subhepatic branch of the portal vein was ligated. Liver washing from the blood elements was performed with oxygenated (95% O_2_ and 5% CO_2_) Krebs-Henseleit buffer (118 mM NaCl, 4.5 mM KCl, 2.75 mM CaCl_2_, 1.19 mM KH_2_PO_4_, 1.18 mM MgSO_4_ and 25 mM NaHCO_3_, pH 7.4 at *t* = 37.8°C) in a volume of 500 ml with a peristaltic pump, creating a flow rate of 2.5–4.0 ml/min/g and a physiological pressure of ~12–15 mm Hg in the portal vein. A recirculating perfusion system was used. In the pleural part, the suprahepatic branch of the vena cava was dissected and the perfusate was removed. The quality of liver perfusion was evaluated based on the organ integrity, uniformity of pale color, and consistency (degree of oedema). After washing blood elements from the liver, liver explantation was performed and isolated perfusion was carried out. MeOH (120 mg/kg) and ALA (20 mg/kg) were sequentially added to the upper perfusate reservoir. Samples were taken every 15 min for an hour with 2 ml perfusate.

### Liver enzyme analysis

Measurements of AST, ALT, and ALP were carried out on an Analyser A25 (Biosystems, Spain) with commercial kits produced by Hospitex Diagnostics (Italy) according to the manufacturer's instructions. The spectrophotometer was calibrated before each enzyme measurement.

### FA measurement by HPLC

All analytical studies were performed on a Dionex Ultimate 3000 (Thermo Fisher, USA), including a quaternary gradient pump, mobile phase degasser, automatic injector and spectrophotometric detector affording continuously variable wavelengths. A reversed phase analytical column, Synergi Hydro-RP, 250 × 4.6 mm, 4 μm sorbent (Phenomenex, USA) was used for the analysis. The mobile phase was water–acetonitrile (50:50, v/v). The detection wavelength was set at 360 nm. The injection volume was 20 μl with a flow rate of 1.0 ml/min. The 0.1% reagent solution was prepared by adding 20 mg of 2,4-dinitrophenylhydrazine hydrochloride (DPH) reagent (TCI, Japan) to 20 ml of acetonitrile and 100 μl of 85% phosphoric acid. To a 20-ml scintillation vial, 50 μL of the cleared sample, 450 μL of deionized water, 2.5 μl of phosphoric acid (85%), and 0.5 ml of the 0.1% DPH reagent were added. Blank samples were prepared by mixing 0.5 ml of the 0.1% DPH reagent with 0.5 ml of deionized water. Formaldehyde was quantitatively converted to the Schiff base in 20 min at 22–24°C. The resultant solution was analyzed by HPLC. Concentrations were quantified according to the equation C (mg/L) = (Sx-Sblank)/14.36^*^20, where 14.36 is the calibration coefficient and 20 is the dilution factor. Measurements of FA were blinded for the HPLC operator.

### MeOH measurements by GC

MeOH content was analyzed on chromatograph Kristall 5000 (Chromatek, Russia) in the following conditions: column Sol-Gel Wax 30^*^0.25^*^0.25, and the flame ionization detector (FID), temperature and injector temperature were set to 220°C. The oven temperature was held for 1 min at 50°C, followed by an increase at 20°C/min to 100°C (hold for 1 min) and ending at 200°C after an increase at a rate of 20°C/min. Nitrogen, hydrogen and air flows were 30, 40, and 400 ml/min, respectively. The injection volume of samples was 1 μl.

### qRT-PCR

RNA concentrations were determined using a Nanodrop ND-1000 spectrophotometer (Isogen Life Sciences). All RNA samples had a 260/280 absorbance ratio between 1.9 and 2.1.

cDNA was obtained by annealing 2 μg of denatured total RNA with 0.1 μg of random hexamers and 0.1 μg of Oligo-dT. The mixture was then incubated with 200 units of Superscript II reverse transcriptase (Invitrogen, USA) for 50 min at 43°C. The qRT-PCR was performed using the iCycler iQ real-time PCR detection system (Bio-Rad, Hercules, CA, USA). For the detection of target genes, the Eva Green master mix (Syntol, Russia) was used according to the manufacturer's instructions. The thermal profile for EVA Green qRT-PCR included an initial heat-denaturing step at 95°C for 3 min and 45 cycles at 95°C for 15 s and an annealing step for 30 s and 72°C for 30 s coupled with fluorescence measurements. Following amplification, the melting curves of the PCR products were monitored from 55 to 95°C to determine the specificity of amplification. Each sample was run in triplicate, and a non-template control was added to each run. Target gene mRNA levels were calculated according to the equation proposed by Pfaffl (2001): ${\text{E}}_{\text{target}}^{\Delta \text{CPtarget}\;\left(\text{sample-reference}\right)}$. PCR efficiency (E) was calculated according to the equation E = 10^(−1/slope)^ based on the standard curves. Target gene mRNA levels were corrected using the corresponding reference genes. Expression analysis was blinded for the qRT-PCR operator. The oligonucleotides used for qRT-PCR are listed in Table S1.

### Measurement of ALDH2 enzymatic activity in mouse brain

The mice were administered ALA (20 mg/kg) concomitantly with 4-MP (10 mg/kg), and 90 min later, the brain total fractions of the mitochondrial and cytoplasmic proteins were separated as described previously (Bunik et al., 2008). The ALDH2 assay was carried out in 200 μl containing 50 mM sodium pyrophosphate buffer pH 8.1, 0.1 mM NAD^+^, 50 mM FA and 20 μl of the brain mitochondrial fraction. ALDH2 and the background reactions (without FA) were recorded in parallel for 20 min at 340 nm in the black plates (25°C). The activities of ALDH2 were normalized for the total protein content (mg) measured by a Pierce BCA Protein Assay kit according to the manufacturer's protocol.

### GSH measurement in mouse brain

GSH was measured using 5,5′-dithio-bis(2-nitrobenzoic acid) (DTNB). Cytoplasmic cell fractions (80 μl) were mixed with 30 μl 10 mM DTNB and 0.2 M Na_2_HPO_4_ to a final volume of 300 μl and 15 min later absorbance was recorded at 412 nm. The concentrations of GSH were normalized for the total protein content (mg) measured by a Pierce BCA Protein Assay kit according to the manufacturer's protocol.

### Synthesis of microarrays

B3 microarray synthesizer (CustomArray, USA) was used for 40 nucleotides-long oligonucleotide probe synthesis on CustomArray ECD 4X2K/12K slides. Synthesis was performed according to the manufacturer's recommendations. Two replicates of total 6,020 unique oligonucleotide probes specific to 2,091 mice gene transcripts were placed on each chip. Chip design was performed using Layout Designer software (CustomArray, USA). For the custom microchip, we used original oligonucleotide probe sequences of the Illumina HT 12 v4 platform.

### Library preparation and hybridization

Complete Whole Transcriptome Amplification WTA2 Kit (Sigma) was used for reverse transcription and library amplification. Manufacturer's protocol was modified by adding to amplification reaction dNTP mix containing biotinylated dUTP, resulting to final proportion dTTP/biotin-dUTP as 5/1. Microarray hybridization was performed according to the CustomArray ElectraSense™ Hybridization and Detection protocol. Hybridization mix contained 2.5 μg of labeleled DNA library, 6X SSPE, 0.05% Tween-20, 20 mM EDTA, 5x Denhardt solution, 100 ng/μl sonicated calf thymus gDNA, 0.05% SDS. Hybridization mix was incubated with chip overnight at 50°C. Hybridization efficiency was detected electrochemically using CustomArray ElectraSense™ Detection Kit and ElectraSense™ 4X2K/12K Reader.

### Statistical analysis

*P*-values were calculated using the Mann–Whitney test or paired Student's *t*-test by R (R: The R Project for Statistical Computing). The boxes of the boxplots represent the median with 25% and 75% percentiles, the bottom whisker is the minimal value, the top whisker is the maximal value, and the outliers are plotted as spots. The barplot heights are the means with standard deviation error bars. Statistical analyses were programmed in R using the Rstudio soft.

## Results

### ALA influence on the MeOH and FA content in mice blood

**Figure 1:**
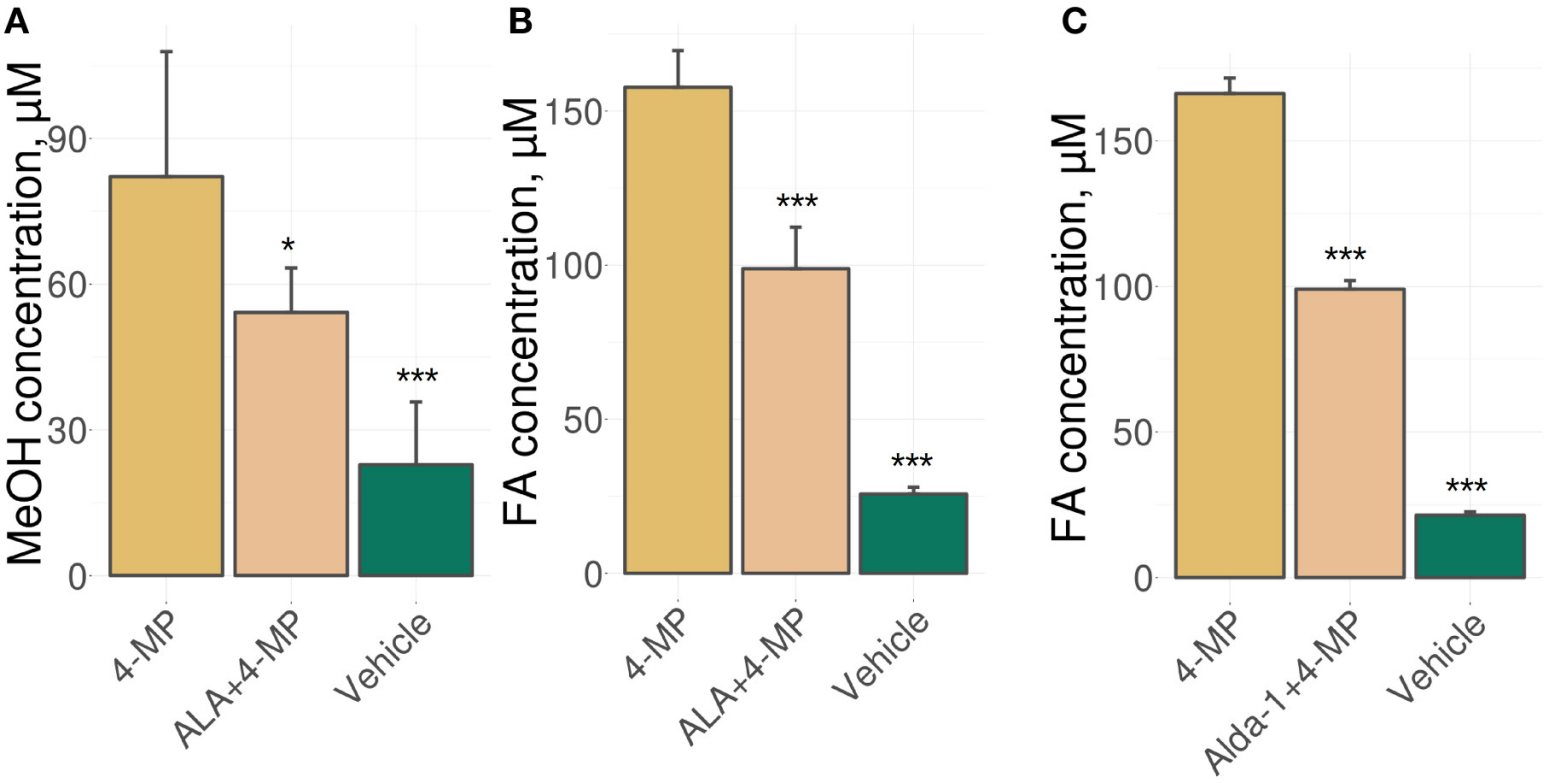
ALA intake decreases the MeOH and FA content in mouse blood. **(A–C)** The mice were administered ALA (20 mg/kg) **(A,B)** or Alda-1 (8.5 mg/kg) **(C)** concomitantly with 4-MP (10 mg/kg), and 90 min later, the MeOH **(A)** and FA **(B,C)** content of the blood was analyzed by GC and HPLC analyses, respectively. The mice were randomly divided into groups of five and received mixture of ALA or Alda-1 and 4-MP in normal saline or only normal saline solution in the control group. The data represent five independent experiments, and standard error bars are indicated. *P*-values (Mann–Whitney *U-*test) are designated by: ^***^P < 0.001; ^*^P < 0.05.

We used an experimental approach in which mice were administered ALA concomitantly with 4-MP and showed that the administration of ALA (20 mg/kg) and 4-MP (10 mg/kg) led to statistically significant reductions of MeOH (Figure 1A) and FA (Figure 1B) content in the blood. The administration of Alda-1, an activator of ALDH2 (Chen et al., 2008; Perez-Miller et al., 2010), also lowered FA in mouse blood (Figure 1C). The results raise questions on the mechanism of ALA action on MeOH and FA metabolism. Because ALA is a multifunctional natural agent with antioxidative properties (Fujita et al., 2008; Kamarudin et al., 2014; Shi et al., 2016), we tested ascorbic acid and tocopherol, known natural antioxidants (Halliwell, 2006), for their ability to influence MeOH and FA metabolism in mice. Surprisingly, there was no difference in the serum levels of MeOH and FA between the mice treated with ascorbic acid (200 mg/kg) or tocopherol (100 mg/kg) and the control groups (Figure S1).

Thus, we concluded that the ability of ALA to exert a diminishing effect on the metabolic content of MeOH and FA does not appear to be directly related to its antioxidant properties.

### Investigation of the ALA effect on hepatic detoxification function in a perfused rat liver model

**Figure 2:**
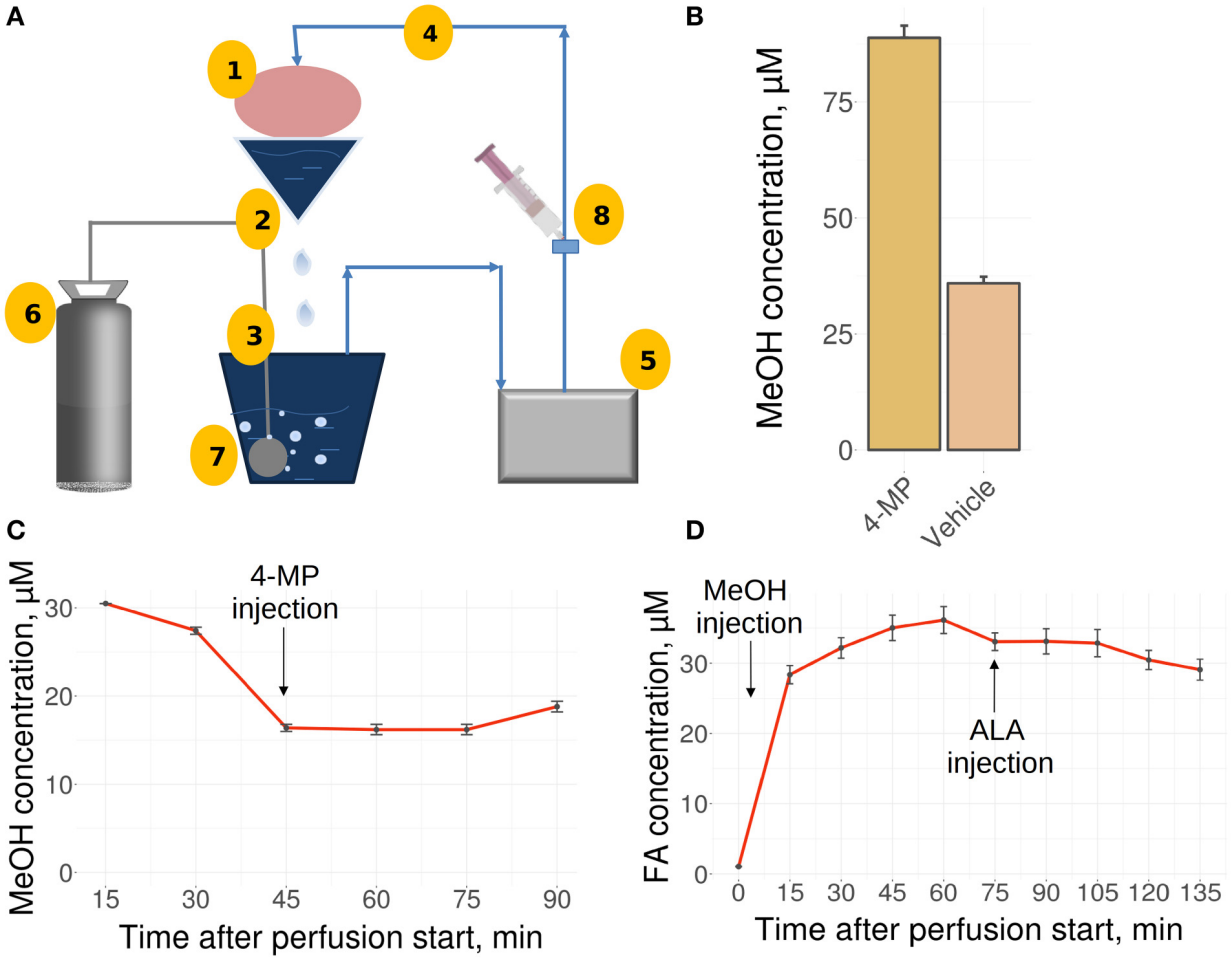
The perfused rat liver model. **(A)** Scheme of the reperfusion system. Parts designated as follows: 1, reperfused rat liver; 2, upper perfusion tank; 3, lower perfusion tank; 4, tube; 5, peristaltic pump; 6, carbogen gas cylinder; 7, oxygenator; 8, injection site. Arrows indicate directions of the buffer flow. **(B)** MeOH in the rat liver before its isolation and perfusion and 30 min after administration of 4-MP (10 mg/kg) to the portal vein. Data shown as the mean of three measurements ± *SD, n* = 3. **(C)** MeOH content in the perfusate samples before and after the administration of 4-MP (10 mg/kg). **(D)** FA concentration in the perfusate samples after MeOH (120 mg/kg) and ALA (20 mg/kg) administration. Data shown as the mean of three measurements ± *SD*.

The liver is the main site for the synthesis of ALA (Mayr et al., 2014; Cronan, 2016) as well as MeOH metabolism, and it could be assumed that the liver is the organ of MeOH generation and FA content control in mammals. To confirm these assumptions, we used a perfused rat liver model (Figure 2A) that preserved the biosynthetic activity of hepatic cells both immediately after organ isolation (Table S2) and during subsequent manipulations (Table S3). When the liver was still connected to the gastrointestinal tract, the introduction of 4-MP into the portal vein led to the accumulation of MeOH in its tissues (Figure 2B). However, the isolated liver, which had ceased all connections with the rest of the body, lost its ability to respond to both 4-MP (Figure 2C) and ALA (Figure 2D).

We concluded that the isolated rat liver failed to generate MeOH and is not sensitive to exogenous ALA.

### ALA influence on mRNA accumulation in mouse organs

**Figure 3:**
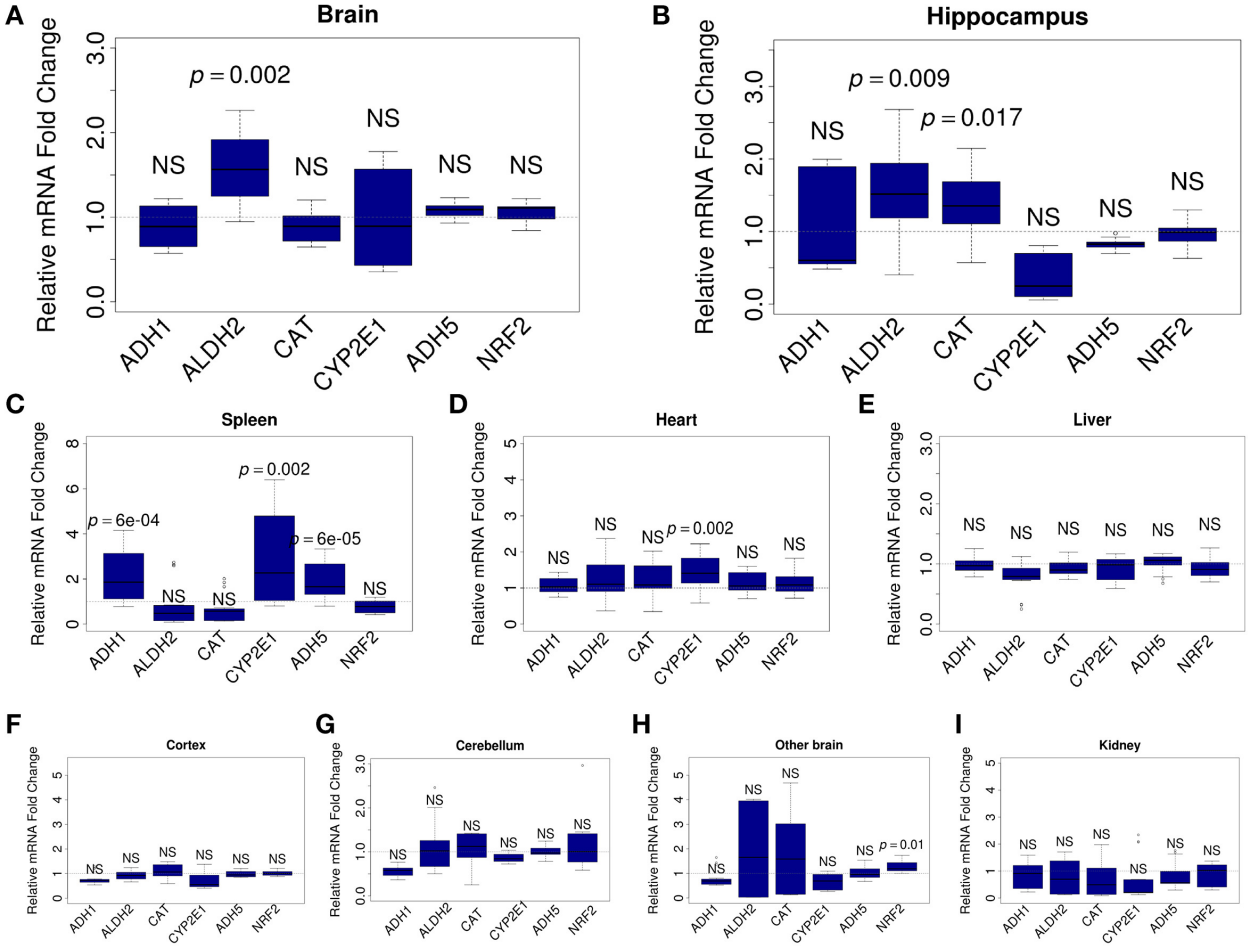
ALA promotes the accumulation of FA-metabolizing gene mRNAs in mouse organs. The mice were administered ALA (20 mg/kg) concomitantly with 4-MP (10 mg/kg) or 4-MP only, and 90 min later, the mRNA content in the whole brain (*n* = 22) **(A)** and brain hippocampus (*n* = 12) **(B)**, spleen (*n* = 12) **(C)**, heart (*n* = 12) **(D)**, and liver (*n* = 22) **(E)**, cortex (*n* = 12) **(F)**, cerebellum (*n* = 12) **(G)**, other brain (*n* = 12) **(H)**, and kidney (*n* = 12) **(I)** was quantified by qRT-PCR. The relative quantities of mRNA after ALA+4-MP intake were normalized to the mRNA levels after 4-MP injection. The data represent two independent experiments. *P*-values (Mann–Whitney *U-*test) vs. 4-MP group are shown; NS- not significant.

To understand which organs react to ALA intake, we analyzed the mRNA content for FA-metabolizing genes. Relative levels of mRNA were quantified by qRT-PCR in mouse brain (whole brain, hippocampus, cortex, cerebellum, and the rest of brain), kidney, spleen, heart, and liver samples. For the analysis, we selected genes that encode key MeOH-metabolizing enzymes (*ADH1, ALDH2, CAT, CYP2E1*, and *ADH5*) and NRF2, which is encoded by the *NFE2L2* gene and is a well-known sensor of oxidative stress (Prasad, 2016). Our analysis showed that ALA intake does not change the mRNA content of the studied genes in the liver (Figure 3E) or the kidney (Figure 3I). The heart responded to ALA with a rise in the content of only *CYP2E1* mRNA (Figure 3D). The spleen (Figure 3C) reacted to ALA by increasing the content of *ADH1, CYP2E1*, and *ADH5* mRNAs. The brain as a whole demonstrated the ability to accumulate the mRNA of genes responsible for FA detoxification (Figure 3A). Apparently, the hippocampus (Figure 3B) is responsible for the responsiveness of the brain to ALA the rest parts of the brain did not reflect any changes (Figures 3F–H).

We concluded that the brain, along with the spleen, are sensitive to the action of ALA and are involved in maintaining a low level of FA in mammals.

### ALA increases GSH synthesis and accelerates FA metabolism by activation of the brain ALDH2 enzyme

**Figure 4:**
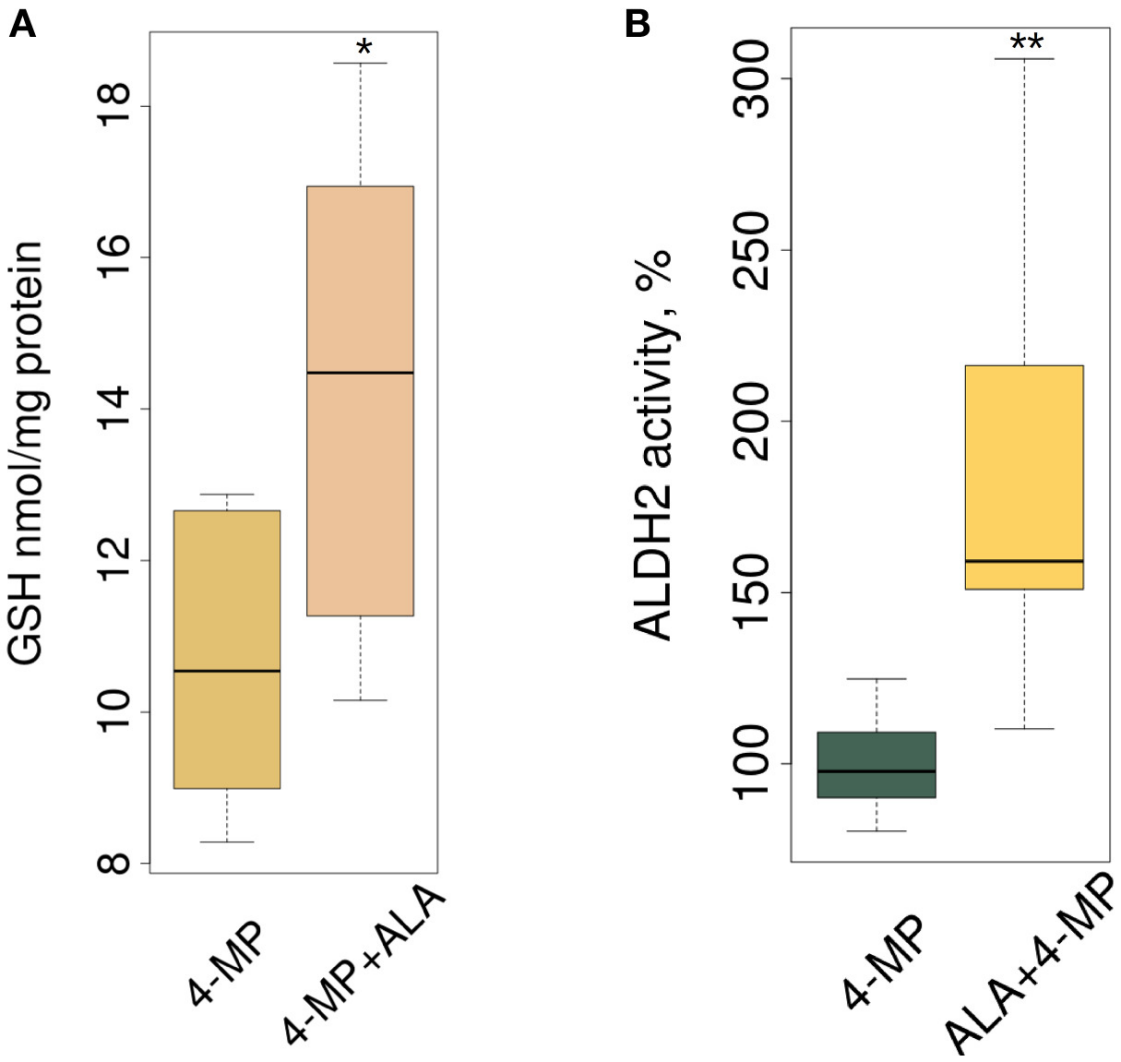
ALA increases GSH synthesis and ALDH2 enzyme activity in mouse brain. The mice were administered ALA (20 mg/kg) concomitantly with 4-MP (10 mg/kg), and 90 min later, the GSH level (*n* = 24) **(A)** and ALDH2 activity (*n* = 24) **(B)** were determined in the cytoplasmic and mitochondrial protein fractions, respectively. The activities of ALDH2 were normalized for total protein (mg) and presented as % of average activity in the control group. Data from 2 independent experiments. *P*-values (Mann–Whitney *U-*test) are designated by: ^**^P < 0.01, ^*^P < 0.05.

Biologically, ALA ameliorates oxidative stress in the mammalian brain, and this mechanism includes increasing the total GSH level (Panigrahi et al., 1996; Suh et al., 2004). Figure 4A, in accordance with our expectations, shows that ALA stimulated GSH accumulation in mouse brain. By stimulating accumulation of the mRNA of detoxifying genes, ALA should also increase the enzymatic activity of their products. To test this assumption, we investigated the enzymatic activity of ALDH2 in the brain extracts of mice after ALA intake. Figure 4B shows the elevated activity of ALDH2 FA-metabolizing enzymes in mouse brains. To exclude the effect of ALA on the specific activity of enzymes, we tested isolated enzymes *in vitro* using various substrates. Figure S2 shows that neither a racemic mixture of ALA or individual isomers (R- and S-ALA) notably affect the activity of ALDH2, ADH5 and ADH1 in liver and brain homogenates *in vitro*.

We concluded that ALA increases the detoxification activity of ALDH2 in the mouse brain without affecting its specific activity.

## Discussion

ALA is widely used as antioxidant in clinical practice (Venigalla et al., 2016) and as a biologically active additive (Ziegler et al., 1995, 1999; Shay et al., 2009).

For the first time, we demonstrated that ALA is able to reduce the level of endogenous FA in mice. Since ALA reduces the content of both FA and MeOH in the blood of mice (Figure 1), we concluded that ALA activated the FA and MeOH oxidation system rather than alternative mechanisms of FA clearance. Expression analysis of the FA-metabolizing gene cluster revealed that ALA intake increased the accumulation of mRNA from FA-metabolizing genes in mouse brains in general, particularly in the mouse hippocampus (*ALDH2, CAT*) and spleen (*ADH1, CYP2E1, ADH5*) (Figure 3). It has been suggested that ALA predominantly ameliorates oxidative stress in the mammalian brain through NRF2 activation (Macias-Barragan et al., 2017). However, our qRT-PCR (Figure 3) and microarray data (Table S4) of mouse brains did not reveal any changes in *NFE2L2* mRNA levels and signaling pathway, indicating that the observed ALA effects on gene expression are mediated by another signaling pathway. On the other hand, our enrichment analysis of differentially expressed genes in the mouse brain in response to ALA and 4-MP vs. 4-MP suggested the potential involvement of the mTOR/S6K1 (Figure S3) and AKT/PI3K (Figure S4) pathways. This possibility is supported by previous reports of effects of ALA on mammalian cells mediated by both of these pathways (Lv et al., 2014; Ma et al., 2016; Dong et al., 2017).

Intriguingly, there was no ALA effect in mouse liver or in a rat liver isolated from the rest of the body (Figure 2). It can be assumed that although the liver is the main organ of ALA synthesis (Hiltunen et al., 2010), ALA targets are located somewhere outside of the liver. Our results showed that the brain is the main organ of ALA effects, and this result is consistent with known data that indicate the ability of ALA to alleviate oxidative stress and affect gene expression in the brain. The mechanism of ALA activation of the *ALDH2* gene in brain cells is unknown.

Although natural antioxidants, namely, vitamins C and E, did not change blood FA (Figure S1), the possibility that the antioxidant properties of ALA are also involved in the activation of the ALDH2 gene in brain cells cannot be excluded. Particular molecules can exhibit cell type-specific antioxidant activities, and their activities can differ depending on the study model that is chosen (Caruso et al., 2017).

It can be assumed that ALA directly or indirectly affects elements of the transcriptional promoter sensitive to acetaldehyde (Ishikawa et al., 2007; Kimura et al., 2009). Our analysis of the ALDH2 promoter (−2,000 … −1 relative to the transcription start site) by UniPROBE (Hume et al., 2015) found 78 distinct transcription factors that bind it *in vitro*. Two of them, SMAD3 and EGR1, are up-regulated in our array data (Table S4), and thus they could be responsible for ALDH2 regulation by ALA.

In the current work, the involvement of ALA in maintaining a low level of FA in mice explains the results of published studies, revealing a relationship between the elevated level of endogenous FA and neurodegeneration in humans (Tang et al., 2013a,b; Tong et al., 2013a, 2017). Our knowledge of the roles of ALA in health and disease permits the customization of existing and future therapeutic and prophylactic modalities.

## Author contributions

YD, AS, and TK conceptualized the topic and designed the experiments. AS and ES performed most of the experiments. NE, VT, AK, GM, IY, and MS conducted some experiments. YD, AS, and TK evaluated the data and drafted the skeleton of manuscript. YD, AS, TK revised and finalized the manuscript. All the authors read and approved the manuscript.

## Conflict of interest statement

The authors declare that the research was conducted in the absence of any commercial or financial relationships that could be construed as a potential conflict of interest.

## Supplementary material

Figure S1 Ascorbic acid and tocopherol do not influence FA level in the mice serum. The mice were administered ascorbate (200 mg/kg, *n* = 3) or tocopherol (100 mg/kg, *n* = 3) concomitantly with 4-MP (10 mg/kg), and 60 min later FA content of the blood was analyzed by HPLC analyses. The mice were randomly divided into groups of three. Data presented as boxplots, where every dot designate one biological replica. NSS, not stastically significant (Student's *t*-test).

Figure S2 ALA failed to alter ALDH2, ADH5 and ADH1 activities *in vitro*. ALDH2 activity was measured in presence of ALA in the mitochondrial protein fraction of liver samples with acetaldehyde (AA) **(A)** or FA **(B)** as substrates. ADH activity was measured in presence of ALA in the cytoplasmic protein fraction of liver or brain samples with EtOH **(C,D)** or MeOH **(E,F)** as substrates. ADH5 activity was measured in the presence of ALA in the cytoplasmic protein fraction of liver samples with FA and glutathione (GSH) as substrates **(G)**. Standard error bars represent the means of triplicate measurements. Asterisks designate data from 2 independent experiments. Activity of the enzymes was calculated as ΔA/min and was presented as % of average activity in the vehicle group.

Figure S3 ALA affects expression of the genes involved in the mTOR signaling pathway. Scheme was colored according to enrichment analysis by DAVID (https://david.ncifcrf.gov/) of the differentially expressed genes in the mice brain treated with ALA and 4-MP vs. 4-MP control. Red color designate the up-regulated genes, blue color—down-regulated. Scheme and coloring were conducted by KEGG Mapper tools (http://www.genome.jp/kegg/tool/map_pathway2.html).

Figure S4 ALA affects expression of the genes involved in the AKT/PI3K signaling pathway. Scheme was colored according to enrichment analysis by DAVID (https://david.ncifcrf.gov/) of the differentially expressed genes in the mice brain treated with ALA and 4-MP vs. 4-MP control. Red color designate the up-regulated genes, blue color—down-regulated. Scheme and coloring were conducted by KEGG Mapper tools (http://www.genome.jp/kegg/tool/map_pathway2.html).

Table S1 Oligonucleotides used for qPCR.

Table S2 Level of ALP, ALT, and AST in the reperfused fluid from the intact isolated rat liver 15 and 30 min after isolation. Data presented as the means ± *SD* of duplicate measurements. ALP, alkaline phosphatase; ALT, alanine aminotransferase; AST, aspartate aminotransferase; ND, not detected.

Table S3 Level of ALP, ALT, and AST in the reperfused fluid from the isolated rat liver 15 min after isolation and 15, 30, 45, and 60 min after MeOH (120 mg/kg) injection. ALP, alkaline phosphatase; ALT, alanine aminotransferase; AST, aspartate aminotransferase; ND, not detected, MeOH, methanol.

Table S4 List of the differentially expressed genes in mice whole brain after ALA treatment. Raw data were quartile normalized and analyzed using the R language. All the genes included in this table had significant changes in their expression compared to the control, with a *P* < 0.05. FC—fold change.
