# Supplementary figures and images for: The Antioxidant Cofactor Alpha-Lipoic Acid May Control Endogenous Formaldehyde Metabolism in Mammals
Source: Front Neurosci. 2017 Dec 1;11:651. doi: 10.3389/fnins.2017.00651 (PMC5717020; doi:10.3389/fnins.2017.00651)

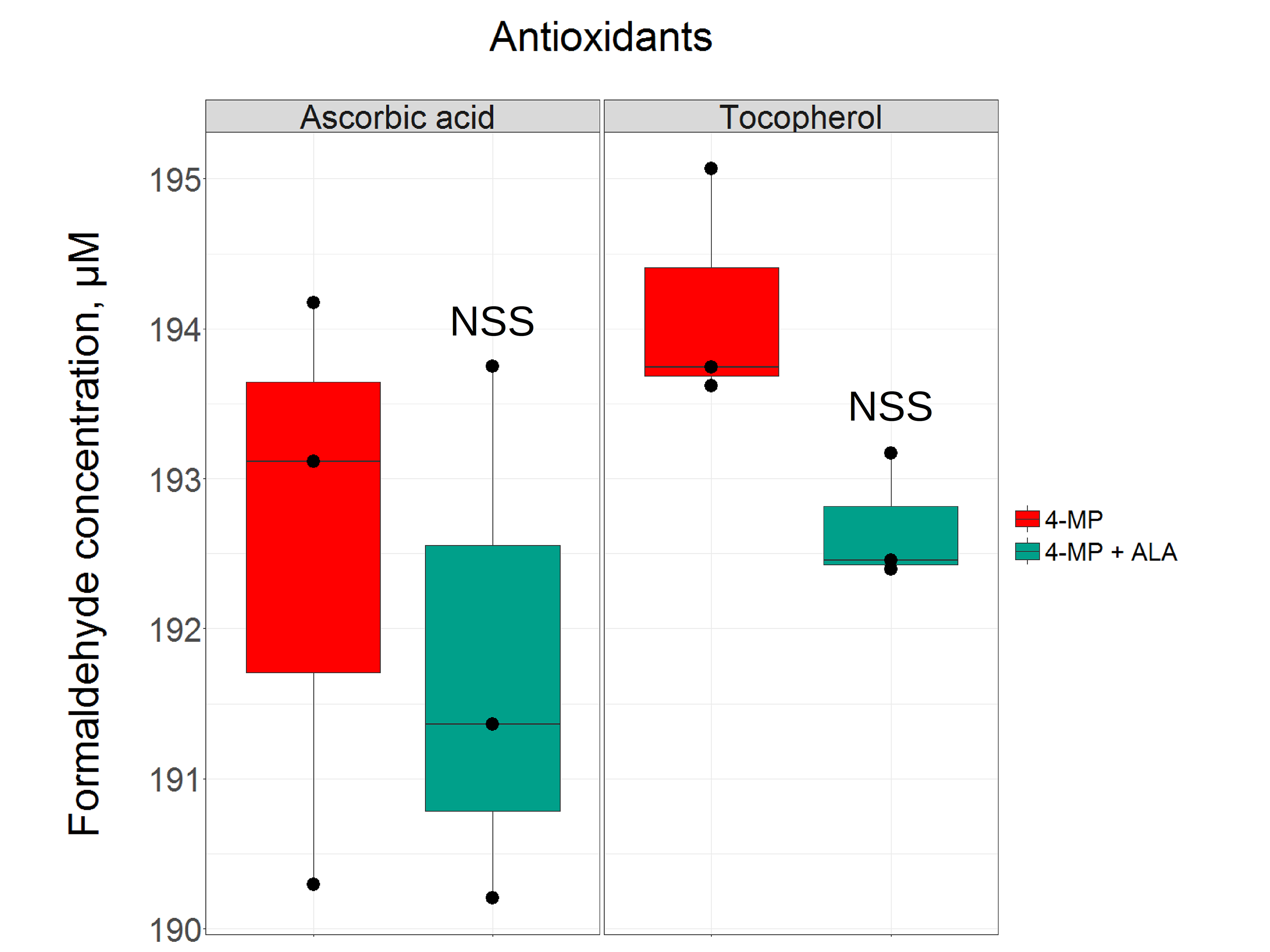

Supplement: Figure S1 — Ascorbic acid and tocopherol do not influence FA level in the mice serum. The mice were administered ascorbate (200 mg/kg, n = 3) or tocopherol (100 mg/kg, n = 3) concomitantly with 4-MP (10 mg/kg), and 60 min later FA content of the blood was analyzed by HPLC analyses. The mice were randomly divided into groups of three. Data presented as boxplots, where every dot designate one biological replica. NSS, not stastically significant (Student's t-test). [file Image1.TIF]

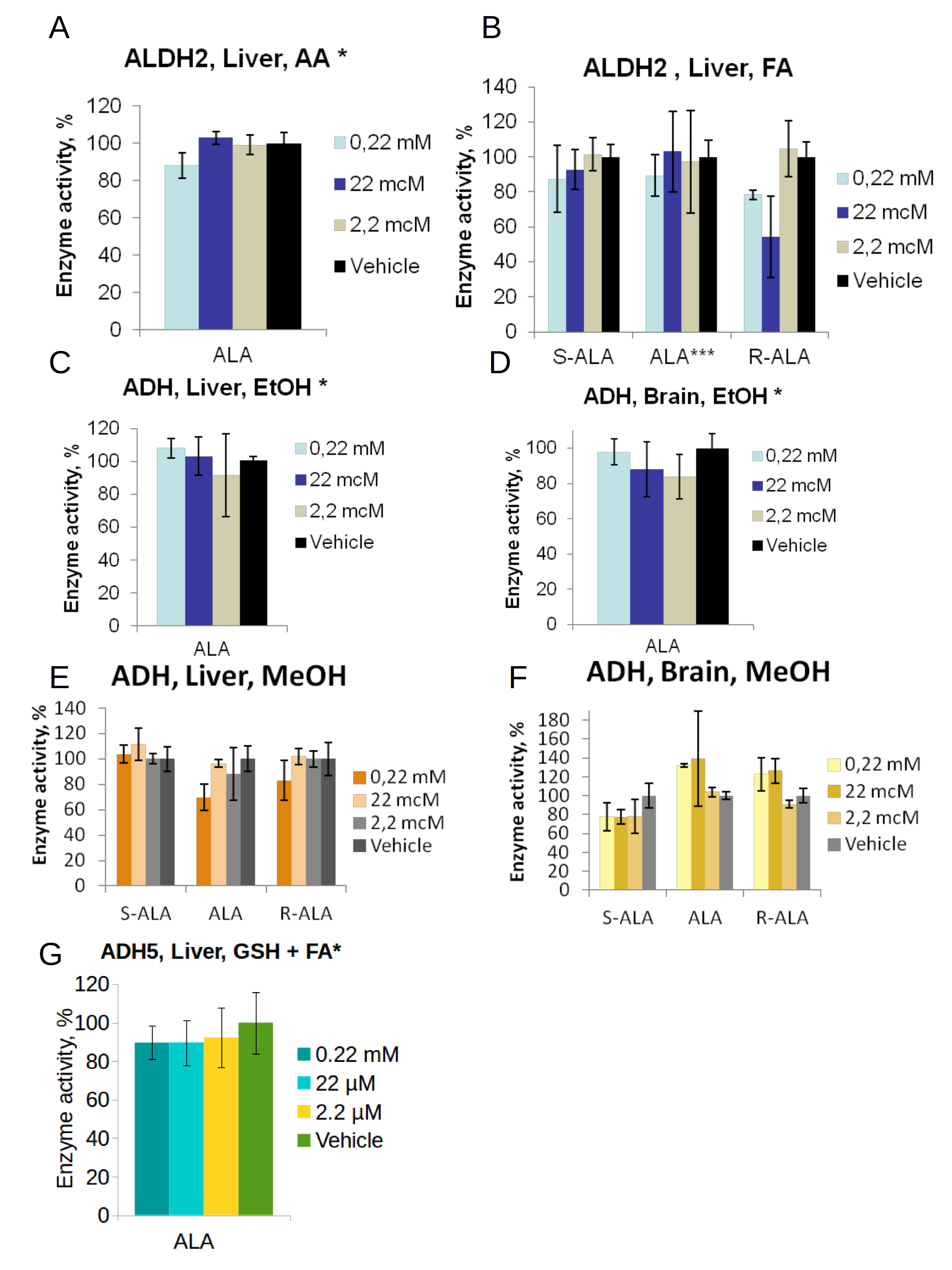

Supplement: Figure S2 — ALA failed to alter ALDH2, ADH5 and ADH1 activities in vitro. ALDH2 activity was measured in presence of ALA in the mitochondrial protein fraction of liver samples with acetaldehyde (AA) (A) or FA (B) as substrates. ADH activity was measured in presence of ALA in the cytoplasmic protein fraction of liver or brain samples with EtOH (C,D) or MeOH (E,F) as substrates. ADH5 activity was measured in the presence of ALA in the cytoplasmic protein fraction of liver samples with FA and glutathione (GSH) as substrates (G). Standard error bars represent the means of triplicate measurements. Asterisks designate data from 2 independent experiments. Activity of the enzymes was calculated as ΔA/min and was presented as % of average activity in the vehicle group. [file Image2.TIF]

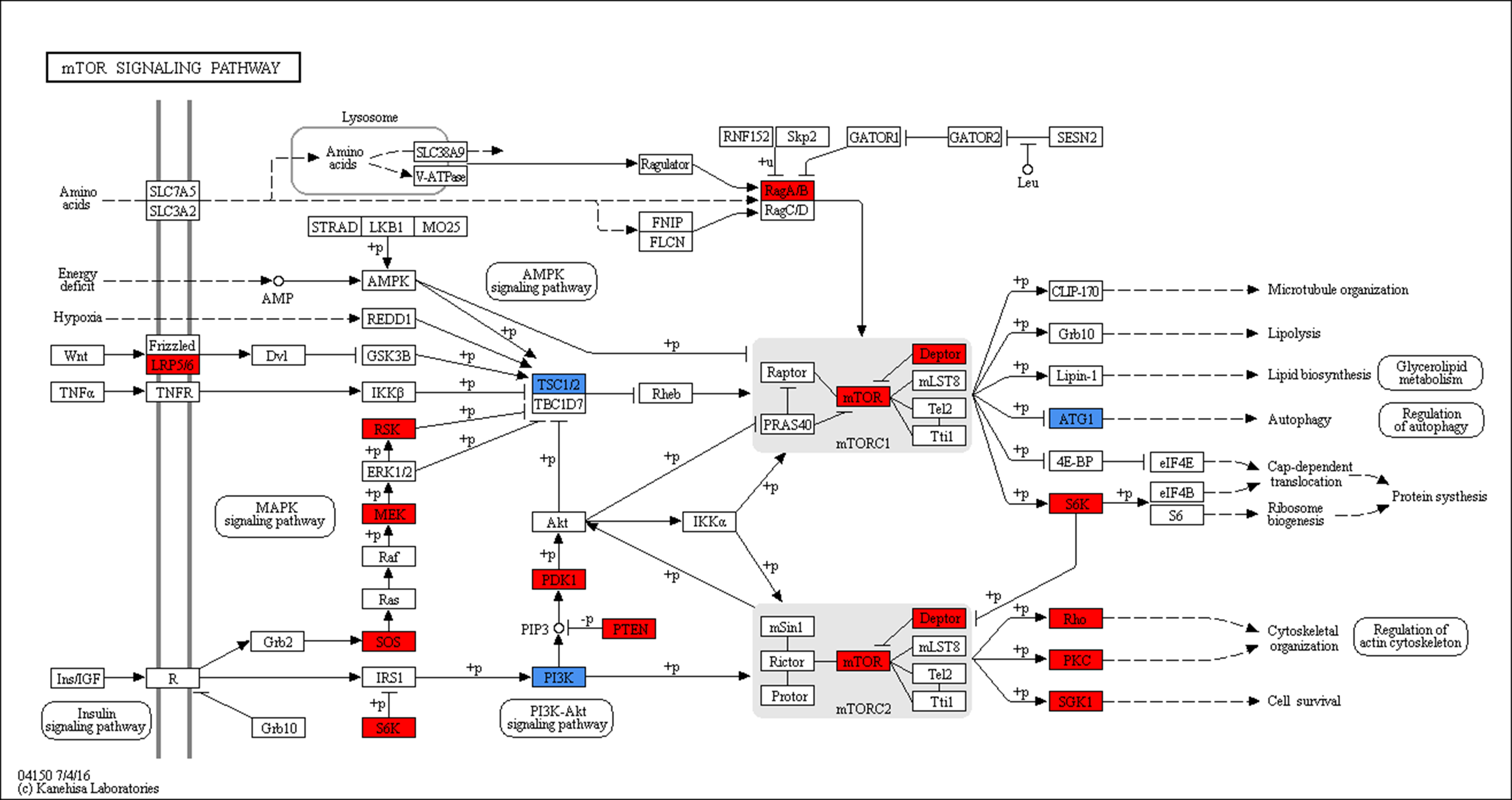

Supplement: Figure S3 — ALA affects expression of the genes involved in the mTOR signaling pathway. Scheme was colored according to enrichment analysis by DAVID (https://david.ncifcrf.gov/) of the differentially expressed genes in the mice brain treated with ALA and 4-MP vs. 4-MP control. Red color designate the up-regulated genes, blue color—down-regulated. Scheme and coloring were conducted by KEGG Mapper tools (http://www.genome.jp/kegg/tool/map_pathway2.html). [file Image3.TIF]

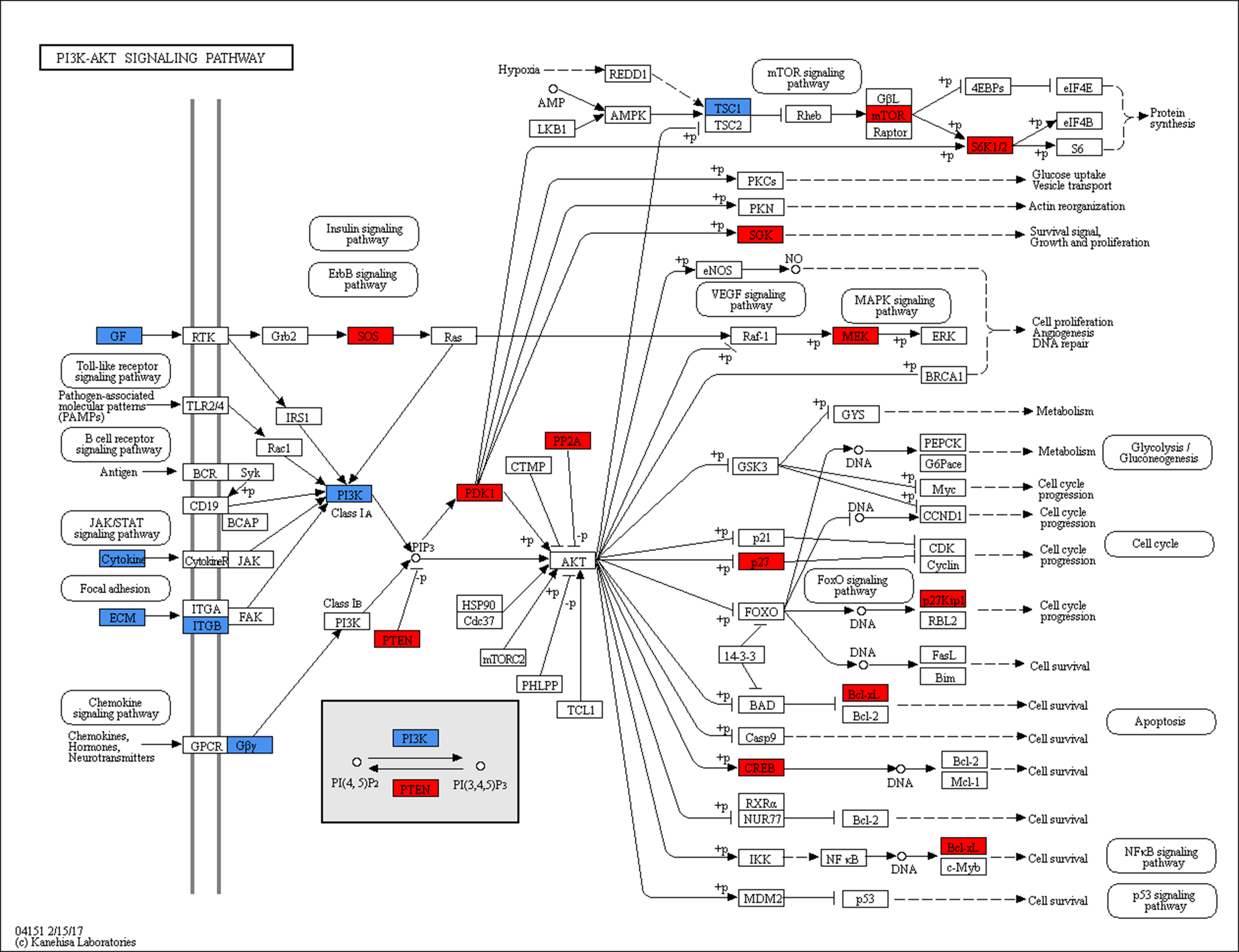

Supplement: Figure S4 — ALA affects expression of the genes involved in the AKT/PI3K signaling pathway. Scheme was colored according to enrichment analysis by DAVID (https://david.ncifcrf.gov/) of the differentially expressed genes in the mice brain treated with ALA and 4-MP vs. 4-MP control. Red color designate the up-regulated genes, blue color—down-regulated. Scheme and coloring were conducted by KEGG Mapper tools (http://www.genome.jp/kegg/tool/map_pathway2.html). [file Image4.TIF]
