# Supplementary material for: The Antioxidant Cofactor Alpha-Lipoic Acid May Control Endogenous Formaldehyde Metabolism in Mammals
Source: Front Neurosci. 2017 Dec 1;11:651. doi: 10.3389/fnins.2017.00651 (PMC5717020; doi:10.3389/fnins.2017.00651)
Supplement: Table S1 — Oligonucleotides used for qPCR. [file Table1.DOC]

| Gene Symbol | Organism | Primers sequence | Annealing temperature, C | Amplicon length. bp |
| --- | --- | --- | --- | --- |
| *ADH1* | Mus musculus | D: GTACCGTCCTGACTTTCTG R: AAGATTAAGGCTGTGATGTGG | 52.2 | 106 |
| *ALDH2* | Mus musculus | D: GACGCCGTCAGCAGGAAAA R: CGCCAATCGGTACAACAGC | 62.9 | 189 |
| *CAT* | Mus musculus | D: TCAGGTTTCTTTCTTGTTCAG R: TGGTCGGTCTTGTAATGG | 52.4 | 138 |
| *CYP2E1* | Mus musculus | D: GAAGAAATTGACAGGGTTATTGG R: GGAAGGGACGAGGTTGATG | 55.6 | 118 |
| *ADH5* | Mus musculus | D: ACTTCATGGGGACTAGCACAT R: AGAAGGCAGACTTTATCCAAAGG | 60 | 100 |
| *NRF2* | Mus musculus | D: AGGAGAGGTAAGAATAAAGTC R: CAAGATACAAGGTGCTGAG | 52.6 | 181 |
