# Supplementary material for: The Antioxidant Cofactor Alpha-Lipoic Acid May Control Endogenous Formaldehyde Metabolism in Mammals
Source: Front Neurosci. 2017 Dec 1;11:651. doi: 10.3389/fnins.2017.00651 (PMC5717020; doi:10.3389/fnins.2017.00651)
Supplement: Table S2 — Level of ALP, ALT, and AST in the reperfused fluid from the intact isolated rat liver 15 and 30 min after isolation. Data presented as the means ± SD of duplicate measurements. ALP, alkaline phosphatase; ALT, alanine aminotransferase; AST, aspartate aminotransferase; ND, not detected. [file Table2.DOC]

**Table S2.** Level of the ALP, ALT and AST in the reperfused fluid from the intact isolated rat liver 15 and 30 minutes after isolation. Data presented as means ± SD of duplicate measurements. ALP - alkaline phosphatase, ALT - alanine aminotransferase, AST - aspartate aminotransferase, ND – not detected.

| Sample | ALP, U/L | ALT, U/L | AST, U/L |
| --- | --- | --- | --- |
| 15 min after isolation | 1.2 ± 0.8 | 3.4 ± 0.6 | 13.9 ± 1.4 |
| 30 min after isolation | ND | 3.7 ± 0.07 | 18.1 |
