# Supplementary material for: The Antioxidant Cofactor Alpha-Lipoic Acid May Control Endogenous Formaldehyde Metabolism in Mammals
Source: Front Neurosci. 2017 Dec 1;11:651. doi: 10.3389/fnins.2017.00651 (PMC5717020; doi:10.3389/fnins.2017.00651)
Supplement: Table S3 — Level of ALP, ALT, and AST in the reperfused fluid from the isolated rat liver 15 min after isolation and 15, 30, 45, and 60 min after MeOH (120 mg/kg) injection. ALP, alkaline phosphatase; ALT, alanine aminotransferase; AST, aspartate aminotransferase; ND, not detected, MeOH, methanol. [file Table3.DOC]

| Sample | ALP, U/L | ALT, U/L | AST, U/L |
| --- | --- | --- | --- |
| 15 min after isolation | ND | 41.3 | 2.9 |
| 15 min after MeOH | ND | 13.3 | 0.3 |
| 30 min after MeOH | ND | 16.3 | 1.4 |
| 45 min after MeOH | ND | 14.2 | 0.6 |
| 60 min after MeOH | ND | 22.2 | 1.6 |

**Table S3.** Level of the ALP, ALT and AST in the reperfused fluid from the isolated rat liver 15 minutes after isolation and 15, 30, 45, 60 minutes after MeOH (120 mg/kg) injection. . ALP - alkaline phosphatase, ALT - alanine aminotransferase, AST - aspartate aminotransferase, ND – not detected, MeOH – methanol.
