# Supplementary material for: The Antioxidant Cofactor Alpha-Lipoic Acid May Control Endogenous Formaldehyde Metabolism in Mammals
Source: Front Neurosci. 2017 Dec 1;11:651. doi: 10.3389/fnins.2017.00651 (PMC5717020; doi:10.3389/fnins.2017.00651)
Supplement: Table S4 — List of the differentially expressed genes in mice whole brain after ALA treatment. Raw data were quartile normalized and analyzed using the R language. All the genes included in this table had significant changes in their expression compared to the control, with a P < 0.05. FC—fold change. [file Table4.DOC]

| Gene Symbol | FC | P-value |
| --- | --- | --- |
| ACTA1 | 2.46 | 0.0382 |
| FGF14 | 2.06 | 0.0006 |
| FAAH | 2.02 | 0.0053 |
| GABRB2 | 1.94 | 0.0007 |
| CDC23 | 1.86 | 0.0079 |
| BMP4 | 1.82 | 0.0148 |
| SLC25A5 | 1.79 | 0.0219 |
| RFK | 1.78 | 0.0334 |
| RAB27A | 1.77 | 0.0106 |
| PTEN | 1.75 | 0.0195 |
| HPCA | 1.73 | 0.0004 |
| SESN3 | 1.72 | 0.0166 |
| CADPS | 1.71 | 0.0181 |
| NTRK2 | 1.71 | 0.0002 |
| KLF10 | 1.70 | 0.0303 |
| HSPA4 | 1.69 | 0.0005 |
| RHOA | 1.67 | 0.0016 |
| NOV | 1.64 | 0.0005 |
| HRH1 | 1.64 | 0.0383 |
| HIF1A | 1.64 | 0.0087 |
| PCNA | 1.63 | 0.0135 |
| CD8A | 1.62 | 0.0059 |
| COL6A1 | 1.60 | 0.0146 |
| SERPING1 | 1.59 | 0.0006 |
| EGR1 | 1.57 | 0.0087 |
| ZEB1 | 1.56 | 0.0060 |
| SOS1 | 1.56 | 0.0248 |
| COL12A1 | 1.55 | 0.0131 |
| S1PR1 | 1.55 | 0.0178 |
| P2RX1 | 1.54 | 0.0035 |
| ANAPC2 | 1.52 | 0.0421 |
| TNIP2 | 1.52 | 0.0118 |
| HNRNPD | 1.52 | 0.0087 |
| ARF3 | 1.52 | 0.0182 |
| MAP2K1 | 1.52 | 0.0133 |
| PRKCB | 1.52 | 0.0024 |
| GABRA1 | 1.52 | 0.0423 |
| PLA2G2F | 1.51 | 0.0110 |
| RYR2 | 1.51 | 0.0083 |
| SMAD3 | 1.51 | 0.0035 |
| CD14 | 1.51 | 0.0207 |
| SCG2 | 1.50 | 0.0373 |
| GRIA2 | 1.49 | 0.0033 |
| SIRPA | 1.49 | 0.0092 |
| CTNNB1 | 1.49 | 0.0220 |
| NOD2 | 1.49 | 0.0236 |
| SPON1 | 1.48 | 0.0236 |
| CREB3 | 1.48 | 0.0037 |
| CCL11 | 1.48 | 0.0299 |
| CHST1 | 1.48 | 0.0109 |
| TM7SF2 | 1.47 | 0.0219 |
| CACNA1I | 1.47 | 0.0126 |
| SLC2A3 | 1.47 | 0.0161 |
| RASGRP1 | 1.46 | 0.0074 |
| FGF23 | 1.46 | 0.0026 |
| ING2 | 1.45 | 0.0317 |
| PSMB5 | 1.45 | 0.0036 |
| PPP2CA | 1.45 | 0.0264 |
| MRC2 | 1.45 | 0.0186 |
| TCF19 | 1.44 | 0.0141 |
| IGFBP4 | 1.44 | 0.0106 |
| SETX | 1.43 | 0.0111 |
| GNA15 | 1.42 | 0.0129 |
| PLD2 | 1.42 | 0.0342 |
| GDF9 | 1.42 | 0.0003 |
| SUMO1 | 1.42 | 0.0104 |
| NLRP12 | 1.42 | 0.0056 |
| GDF1 | 1.41 | 0.0263 |
| CACNG7 | 1.41 | 0.0143 |
| ENC1 | 1.41 | 0.0043 |
| NCOR1 | 1.41 | 0.0208 |
| RHOB | 1.40 | 0.0251 |
| CARTPT | 1.40 | 0.0054 |
| CNR1 | 1.40 | 0.0068 |
| PON3 | 1.39 | 0.0042 |
| MAPK12 | 1.39 | 0.0088 |
| CYP4X1 | 1.39 | 0.0051 |
| PROK1 | 1.39 | 0.0106 |
| FADS1 | 1.38 | 0.0085 |
| RAP1B | 1.38 | 0.0120 |
| PRKAR1B | 1.38 | 0.0265 |
| VDAC2 | 1.38 | 0.0439 |
| HP | 1.37 | 0.0311 |
| PRDX1 | 1.37 | 0.0128 |
| PRNP | 1.37 | 0.0231 |
| FLT3 | 1.37 | 0.0011 |
| NQO2 | 1.37 | 0.0026 |
| RHOC | 1.37 | 0.0063 |
| RRAGA | 1.37 | 0.0083 |
| PDPK1 | 1.37 | 0.0119 |
| TRIL | 1.36 | 0.0223 |
| CDH11 | 1.36 | 0.0155 |
| HPRT | 1.36 | 0.0057 |
| FYN | 1.36 | 0.0354 |
| SGK1 | 1.35 | 0.0124 |
| COL11A1 | 1.34 | 0.0029 |
| VDAC1 | 1.34 | 0.0403 |
| CYP46A1 | 1.34 | 0.0340 |
| PPP2CB | 1.33 | 0.0390 |
| GNAI3 | 1.33 | 0.0357 |
| STX1A | 1.33 | 0.0480 |
| LTBP1 | 1.33 | 0.0179 |
| GPRC5B | 1.33 | 0.0044 |
| MAPK13 | 1.32 | 0.0247 |
| RPS6KB1 | 1.32 | 0.0069 |
| CACNB1 | 1.32 | 0.0051 |
| PLCH2 | 1.32 | 0.0192 |
| DEPTOR | 1.32 | 0.0079 |
| PARP6 | 1.31 | 0.0461 |
| CNBP | 1.31 | 0.0457 |
| CYP20A1 | 1.31 | 0.0096 |
| CALM1 | 1.31 | 0.0048 |
| ZKSCAN3 | 1.30 | 0.0189 |
| GRIA1 | 1.30 | 0.0052 |
| RB1CC1 | 1.29 | 0.0115 |
| PSMB7 | 1.29 | 0.0008 |
| POLR3B | 1.29 | 0.0235 |
| GJA1 | 1.29 | 0.0326 |
| PID1 | 1.29 | 0.0046 |
| GGT7 | 1.29 | 0.0143 |
| MTOR | 1.29 | 0.0165 |
| NOTCH1 | 1.29 | 0.0042 |
| PDK4 | 1.28 | 0.0442 |
| GNA12 | 1.28 | 0.0007 |
| MCTP1 | 1.28 | 0.0362 |
| ADRA2C | 1.28 | 0.0199 |
| PLS3 | 1.28 | 0.0271 |
| GLRX2 | 1.27 | 0.0022 |
| P2RY12 | 1.27 | 0.0080 |
| RAC2 | 1.27 | 0.0292 |
| DMTN | 1.27 | 0.0387 |
| CHST4 | 1.27 | 0.0041 |
| BAK1 | 1.26 | 0.0329 |
| CACNB3 | 1.26 | 0.0092 |
| F3 | 1.26 | 0.0351 |
| PSMB1 | 1.25 | 0.0270 |
| VAV3 | 1.25 | 0.0449 |
| PNPLA8 | 1.25 | 0.0097 |
| CYCS | 1.25 | 0.0216 |
| TBC1D23 | 1.25 | 0.0310 |
| ZP3 | 1.25 | 0.0111 |
| CDKN1B | 1.25 | 0.0467 |
| LRP6 | 1.25 | 0.0179 |
| PRKCH | 1.24 | 0.0058 |
| TUBA8 | 1.24 | 0.0124 |
| CDKN2C | 1.23 | 0.0371 |
| SETD6 | 1.23 | 0.0444 |
| CYP11B1 | 1.23 | 0.0181 |
| PLIN5 | 1.23 | 0.0041 |
| P2RY1 | 1.22 | 0.0268 |
| PSMC2 | 1.22 | 0.0322 |
| SNTA1 | 1.22 | 0.0092 |
| LAMC1 | 1.21 | 0.0094 |
| PYCR1 | 1.21 | 0.0272 |
| BCL9 | 1.20 | 0.0201 |
| HDAC7 | 1.20 | 0.0197 |
| AKR1B3 | 1.20 | 0.0477 |
| BCL2L1 | 1.20 | 0.0234 |
| PFKM | 1.19 | 0.0376 |
| PAK1IP1 | 1.19 | 0.0188 |
| CDC42 | 1.19 | 0.0407 |
| IMPG2 | 1.18 | 0.0284 |
| PSMC4 | 1.18 | 0.0049 |
| RPS6KA3 | 1.17 | 0.0103 |
| NLRP4C | 1.17 | 0.0453 |
| GNAO1 | 1.17 | 0.0455 |
| TUBB2B | 1.16 | 0.0143 |
| ANGPTL1 | 1.15 | 0.0494 |
| C8B | 1.15 | 0.0257 |
| SHC3 | 1.14 | 0.0192 |
| NUPR1 | 1.13 | 0.0465 |
| CCL25 | 1.13 | 0.0177 |
| CDH5 | 1.11 | 0.0343 |
| BCL6 | 1.10 | 0.0336 |
| TH | 0.89 | 0.0152 |
| ANAPC7 | 0.89 | 0.0363 |
| GNAZ | 0.88 | 0.0192 |
| CIITA | 0.87 | 0.0174 |
| ANAPC4 | 0.86 | 0.0249 |
| TCF12 | 0.86 | 0.0159 |
| GRM1 | 0.85 | 0.0388 |
| AKR1C6 | 0.85 | 0.0375 |
| P4HB | 0.85 | 0.0080 |
| EEF2 | 0.85 | 0.0241 |
| CUEDC2 | 0.84 | 0.0074 |
| CD2 | 0.84 | 0.0345 |
| GABRB1 | 0.83 | 0.0048 |
| POLR3D | 0.82 | 0.0057 |
| MMP23 | 0.82 | 0.0048 |
| ADCY4 | 0.82 | 0.0349 |
| RARRES2 | 0.82 | 0.0141 |
| ITGB2 | 0.81 | 0.0212 |
| IFNG | 0.81 | 0.0341 |
| GPC4 | 0.81 | 0.0404 |
| DLG1 | 0.81 | 0.0122 |
| ANGPT2 | 0.81 | 0.0366 |
| MVD | 0.81 | 0.0314 |
| HRH4 | 0.80 | 0.0317 |
| COL23A1 | 0.80 | 0.0356 |
| MMP21 | 0.80 | 0.0211 |
| AVP | 0.80 | 0.0210 |
| PRKCZ | 0.80 | 0.0000 |
| PGF | 0.80 | 0.0115 |
| ELF1 | 0.79 | 0.0124 |
| NFAT5 | 0.79 | 0.0130 |
| CH25H | 0.78 | 0.0340 |
| LTBP3 | 0.78 | 0.0211 |
| APH1A | 0.78 | 0.0309 |
| CDH19 | 0.78 | 0.0248 |
| NCSTN | 0.78 | 0.0350 |
| IL17B | 0.78 | 0.0033 |
| CD27 | 0.78 | 0.0117 |
| MCRS1 | 0.77 | 0.0009 |
| TAT | 0.77 | 0.0471 |
| FGR | 0.77 | 0.0085 |
| CD4 | 0.76 | 0.0043 |
| P2RX2 | 0.76 | 0.0488 |
| S100A8 | 0.76 | 0.0210 |
| AGER | 0.76 | 0.0159 |
| PLA2G5 | 0.76 | 0.0182 |
| MCTP2 | 0.75 | 0.0321 |
| PIK3R2 | 0.75 | 0.0001 |
| FGF17 | 0.74 | 0.0039 |
| HRH3 | 0.74 | 0.0370 |
| MAST2 | 0.74 | 0.0011 |
| CFB | 0.73 | 0.0079 |
| EPHB6 | 0.73 | 0.0216 |
| LDLR | 0.73 | 0.0360 |
| CDO1 | 0.73 | 0.0199 |
| TNFRSF11B | 0.73 | 0.0057 |
| NOS1 | 0.72 | 0.0182 |
| GNB5 | 0.72 | 0.0044 |
| NQO1 | 0.72 | 0.0022 |
| ERBB4 | 0.72 | 0.0460 |
| ADCY7 | 0.71 | 0.0085 |
| THEMIS2 | 0.71 | 0.0167 |
| LAMA5 | 0.71 | 0.0206 |
| GNB4 | 0.71 | 0.0120 |
| PCX | 0.71 | 0.0040 |
| TBXAS1 | 0.70 | 0.0020 |
| CYP7A1 | 0.70 | 0.0253 |
| FGF22 | 0.70 | 0.0327 |
| DIABLO | 0.70 | 0.0249 |
| PPP2R5C | 0.70 | 0.0030 |
| NOTCH2 | 0.70 | 0.0087 |
| ERCC6 | 0.69 | 0.0033 |
| TSC1 | 0.69 | 0.0440 |
| APOC1 | 0.69 | 0.0013 |
| ECM1 | 0.68 | 0.0079 |
| BRD4 | 0.68 | 0.0034 |
| NFATC3 | 0.67 | 0.0074 |
| LGALS9 | 0.67 | 0.0227 |
| SMAD6 | 0.67 | 0.0481 |
| CAPNS2 | 0.67 | 0.0041 |
| TNFRSF14 | 0.66 | 0.0023 |
| TRAT1 | 0.66 | 0.0179 |
| ACVRL1 | 0.66 | 0.0494 |
| MAP2K5 | 0.66 | 0.0014 |
| KLRG1 | 0.66 | 0.0191 |
| AREG | 0.66 | 0.0244 |
| PLCE1 | 0.66 | 0.0224 |
| NCF2 | 0.66 | 0.0452 |
| HTR1B | 0.66 | 0.0380 |
| ADPRHL2 | 0.65 | 0.0051 |
| IL3 | 0.65 | 0.0177 |
| HAT1 | 0.65 | 0.0325 |
| CD59B | 0.64 | 0.0189 |
| DAB2IP | 0.64 | 0.0111 |
| GDF6 | 0.63 | 0.0067 |
| GNG8 | 0.63 | 0.0027 |
| FGF8 | 0.63 | 0.0350 |
| EDN2 | 0.62 | 0.0043 |
| ADCYAP1 | 0.61 | 0.0324 |
| NFATC4 | 0.61 | 0.0051 |
| COL4A3 | 0.61 | 0.0030 |
| NRG1 | 0.60 | 0.0028 |
| ALOX5AP | 0.59 | 0.0126 |
| LEFTY2 | 0.59 | 0.0362 |
| CXCL12 | 0.58 | 0.0051 |
| CAPN9 | 0.58 | 0.0009 |
| ULK2 | 0.57 | 0.0447 |
| ITGB6 | 0.56 | 0.0002 |
| JPH1 | 0.56 | 0.0069 |
| ECM2 | 0.56 | 0.0330 |
| CFH | 0.56 | 0.0009 |
| RND2 | 0.54 | 0.0496 |
| ATP1B1 | 0.53 | 0.0226 |
| PLCB4 | 0.53 | 0.0179 |
| DUSP10 | 0.51 | 0.0001 |
| DYNLL2 | 0.49 | 0.0423 |
| CD70 | 0.47 | 0.0031 |
| ATF7 | 0.45 | 0.0483 |
| BTC | 0.44 | 0.0104 |
| CAPN7 | 0.42 | 0.0005 |
| DMD | 0.41 | 0.0333 |
